# Hypertension Identification using Mobile Health Outreach Project (HI HOPE): A Pilot Study

**DOI:** 10.1007/s10900-026-01605-7

**Published:** 2026-08-10

**Authors:** Rose Baumann, Jonathan D Kirsch, Kimberly Clinch, Maggie Eckerstorfer, Rishi Sharma, Ritwick Banjeree, Shahidali Jaffer, Haitham M Hussein

**Affiliations:** 1 Department of Medicine, University of Minnesota, 420 Delaware St SE, MMC 741, Minneapolis, MN 55455 USA; 2 Office of Academic Clinical Affairs, University of Minnesota, 426 Church St SE, Minneapolis, MN 55455 USA; 3 University of Minnesota Medical School, 420 Delaware St SE, Minneapolis, MN 55455 USA; 4 Department of Neurology, Mayo Clinic, 200 First St SW, Rochester, MN 55902 USA; 5 Department of Neurology, University of Minnesota, 420 Delaware St SE, MMC 295, Minneapolis, MN 55455 USA

**Keywords:** Mobile clinic, Hypertension, Blood pressure, Health disparities, Blood pressure monitor, Access to primary care

## Abstract

Racial disparities in the management of hypertension, a risk factor for cardiovascular disease, are prevalent. Mobile health clinics, including the University of Minnesota (UMN) Mobile Health Initiative (MHI), seek to mitigate these disparities by providing underserved individuals with access to screening for elevated blood pressure. However, the impact of this process on subsequent care, including diagnosis and management of hypertension, is unknown. Individuals with elevated blood pressure at UMN MHI screening events were provided with a free self-monitoring blood pressure device (SMBP) and a follow-up phone call two weeks after the event. The primary outcome was the percentage of participants reached with a follow-up call; secondary outcomes included SMBP usage and arrangement of primary care follow-up. Fifty-six participants were enrolled, with a mean age of 57 ± 14 years, half were women, and more than 80% self-identified as non-white. Thirty-seven (66%) participants were successfully reached with a follow-up phone call. The average time to reach participants was 36 days. Fifteen (41%) required more than one phone call attempt. Of the 37 participants who were successfully contacted, 33 (89%) had used SMBP, 27 (82%) reported using the SMBP at least weekly, and 21 (57%) had contacted their primary care doctor by the time of the follow-up phone call. Participant use of SMBP devices was high; however, contacting individuals for follow-up via phone calls was challenging. Distribution of SMBP devices and follow-up phone calls may be a useful way for mobile health clinics to help individuals with hypertension improve their management through increased awareness and by creating a bridge to primary care follow-up.

## Background

Hypertension is the most prevalent risk factor for stroke and cardiovascular disease [1, 2]. Studies have consistently demonstrated racial disparities in hypertension prevalence and control. In a study that used data from the NHANES 2011–2016 survey years and included 16,103 adult participants, the prevalence of hypertension was lower among white participants (45.7%) compared with African American participants (59%), who had the highest proportion of unmet treatment goals for blood pressure control (63.8%) [3]. In our state of Minnesota, after adjusting for age differences, 35% of African American Minnesotans reported high blood pressure (BP), compared with 28% of Minnesotans overall in 2023 [4], and the rate of uncontrolled hypertension is highest in African American and Hmong American patients [5].

Mobile health clinics aim to enhance access to healthcare for individuals facing health disparities, including those affected by hypertension. Often, these clinics do not provide longitudinal care for the conditions they encounter, but instead seek to identify, educate, and refer patients accordingly. Evidence exists that mobile health clinics in the United States facilitate healthcare access to racial minorities and individuals who do not have consistent primary care [6, 7]. However, the long-term benefits of these encounters to participants’ overall health are unknown.

The University of Minnesota (UMN) Mobile Health Initiative (MHI) provides health screening services at community-based health-focused events, which are typically organized in collaboration with local community organizations. Every month, there are multiple events in the Twin Cities metro area and in rural Minnesota. The MHI routinely visits community partners but attends specific community events at inconsistent intervals. Individuals typically attend the MHI clinic once, with no intention of follow-up visits, as participation is voluntary and no current guidelines for such screenings exist. The majority of individuals seen by MHI are from underserved populations, including rural communities as well as racial/ethnic minority communities, including African American, Hispanic/Latino, and multiple refugee groups, such as Hmong, Somali, and Afghan.

The standard MHI workflow includes checking BP and hemoglobin A1c using point-of-care devices. MHI also provides eye exams and free glasses, basic dental care, cancer screening, counseling on healthy lifestyles, medication management, and assistance with health-related questions. Since hypertension screening is a standard component of MHI clinic visits, our team has identified many individuals with elevated BP and provided relevant counseling and education over the years. While the subsequent phase of care following the MHI visit remains unknown, the concern is that many individuals do not get optimal care. This uncertainty, combined with our desire to improve the transition of care from the MHI clinic to primary care, led to the creation of this project.

Hypertension Identification using mobile Health Outreach ProjEct (HI HOPE) is a proof-of-concept pilot project that seeks to evaluate a new model of care that integrates self-monitoring of blood pressure (SMBP) devices and follow-up calls into the workflow of our MHI clinic. Prior studies have shown that using an SMBP device alone reduces blood pressure (BP) and improves medication adherence compared with standard care [8, 9]. However, very few studies have been conducted to assess the efficacy of SMBP in reducing BP in racial minority populations [9]. Furthermore, this effect has not been studied in the setting of mobile clinics.

## Methods

HI HOPE is a single-arm pilot project to evaluate the feasibility of a new MHI workflow, which adds two elements to the current workflow: (1) the provision of a free SMBP device to individuals with elevated BP at screening and (2) a follow-up phone call by the MHI team.

Study participants were recruited from MHI health screening events between 2022 and 2023. Inclusion criteria were age ≥ 18 years, elevated BP (defined as systolic blood pressure (SBP) ≥ 140 mmHg and/or diastolic blood pressure ≥ 90 mmHg), ability of participants to be reached by phone, and participants’ informed consent signatures. The BP cutoff was chosen as a qualifying criterion and not intended to diagnose participants with hypertension. Exclusion criteria included: already having an SMBP device, terminal illness/hospice care, and any medical condition that would prevent a participant from measuring, recording, and/or communicating their BP to the study team.

During BP screening at health screening events, individuals who met the HI HOPE criteria were invited to participate. Those who consented were provided with a free, FDA-approved SMBP device, instructed on its use by a researcher who offered to demonstrate its use at the event, encouraged to use the SMBP device frequently, and educated about hypertension diagnosis and management using printed educational materials in their preferred language [10, 11]. Participants were advised to contact their PCP within 4 weeks of the MHI visit for follow-up if they had a PCP and were offered assistance in finding a PCP if they did not have one.

The HI HOPE workflow also included collecting participants’ telephone numbers and contacting them two weeks after their MHI visit. During the follow-up calls, a scripted interview was conducted. Interview questions addressed the use of the SMBP device, hypertension health literacy, and access to primary care. The study protocol included three attempts at contacting each participant before labeling them as unreachable.

The primary outcome was the percentage of participants successfully contacted by phone after the MHI clinic visit. HI HOPE would be considered feasible if 90% or more of participants were successfully contacted with a follow-up phone call. Secondary outcomes evaluated included (1) the number of participants who used the SMBP device at least once, (2) the frequency of SMBP device usage, (3) the number of participants who attempted to follow up with a primary care provider (PCP) by making at least one phone call to a clinic, (4) the number of participants who need assistance to access primary care, and (5) the number of participants who have scheduled or already attended a primary care visit. The lessons learned from this pilot were intended to inform the design of a more extensive study of a similar methodology.

HI HOPE was an unfunded study. The SMBP devices were purchased from the MHI operations budget, and the study team volunteered to conduct the study. The sample size was determined by the financial resources available to purchase SMBP devices, which we estimated at around 50 devices. HI HOPE was approved by the UMN Institutional Review Board (IRB).

## €Results

Fifty-six participants were recruited over nine MHI outreach events between November 2022 and September 2023. Median BP at enrollment was 145/88 mm Hg. The mean age of participants was 57 ± 14 years, and approximately half were women. The self-reported race/ethnic distribution was as follows: 57% Black/African American, 23% Hispanic, and 20% mixed, white, or unknown. Refer to Table 1 for further demographic information, including insurance status and preferred language. Thirty-two participants (57%) reported a history of hypertension, and 25 of those were on antihypertensive medications. Of the 56 enrolled participants, 37 (66%) reported having an established PCP before the event, with a median time of two months since their last visit.

**Table 1:** Participant characteristics

Participant Characteristics	Value
Total Participants	56
Age (in years)	
Mean	57 ± 14
Female	29
Male	27
Race and Ethnicity (self-reported)	
Black/African American	32 (57%)
Country of Origin (self-reported)	
Somalia	9 (16%)
United States	12 (21%)
Nigeria	2 (4%)
Liberia	2 (4%)
Not specified	7 (13%)
Hispanic/Latino	13 (23%)
White	5 (9%)
Mixed or unknown	6 (11%)
Preferred Language	
English	27
Spanish	11
Somali	8
English and Somali	5
Lao	1
Not indicated	4
Insurance status	
Private insurance	17 (30%)
Medicare	10 (18%)
Medicaid	7 (13%)
Uninsured	12 (21%)
History of HTN	32 (57%)
History of HTN + Antihypertensive Medication	25 (45%)
Previously Established PCP	37 (66%)

Follow-up calls were attempted for each of the 53 participants with a valid phone number (two participants had invalid phone numbers, and one did not have a number listed). Thirty-seven participants were successfully contacted, representing 66% of the total sample and 70% of the participants with valid phone numbers. Twenty-two participants were available at the first-attempt follow-up phone calls, with the rest requiring multiple attempts (Table 2). The average time to reach participants was 36 days. Ten of the successfully reached participants preferred a language other than English and thus required coordination of interpreter services for the follow-up call. Sixteen participants (30%) with valid phone numbers did not answer a follow-up phone call after three attempts.

**Table 2:** Follow-up Phone Call Results

Follow-up Phone Call Results	*n* (%)
Total participants	56
Participants with a valid phone number	53 (95%)
Successfully contacted	37 (66%)
First attempt follow-up call	22 (59%)
Second attempt follow-up call	7 (19%)
Third attempt follow-up call	7 (19%)
*Fourth attempt follow-up call	1 (3%)

*One participant had a fourth attempt by mistake

Of the 37 participants reached with a follow-up phone call, 33 (89%) had used the SMBP. Of these, 27 participants (82%) established regular use of the device after receiving it, defined as at least weekly use; see Figure for a further breakdown of use. The median reported BP, as recorded by SMBP at the time of the follow-up call, was 138/85. Three participants reported difficulties using the SMBP. The first realized their high readings were due to holding their arm incorrectly and, after appropriate positioning, recognized that the readings were corrected. The second reported the display of letters instead of numbers. The third reported values that were likely erroneous (300/200 or 50/0). Six participants reported that the BP values of SMBP provided by HI HOPE were higher or lower than those from another device they used.

**Fig. 1:**
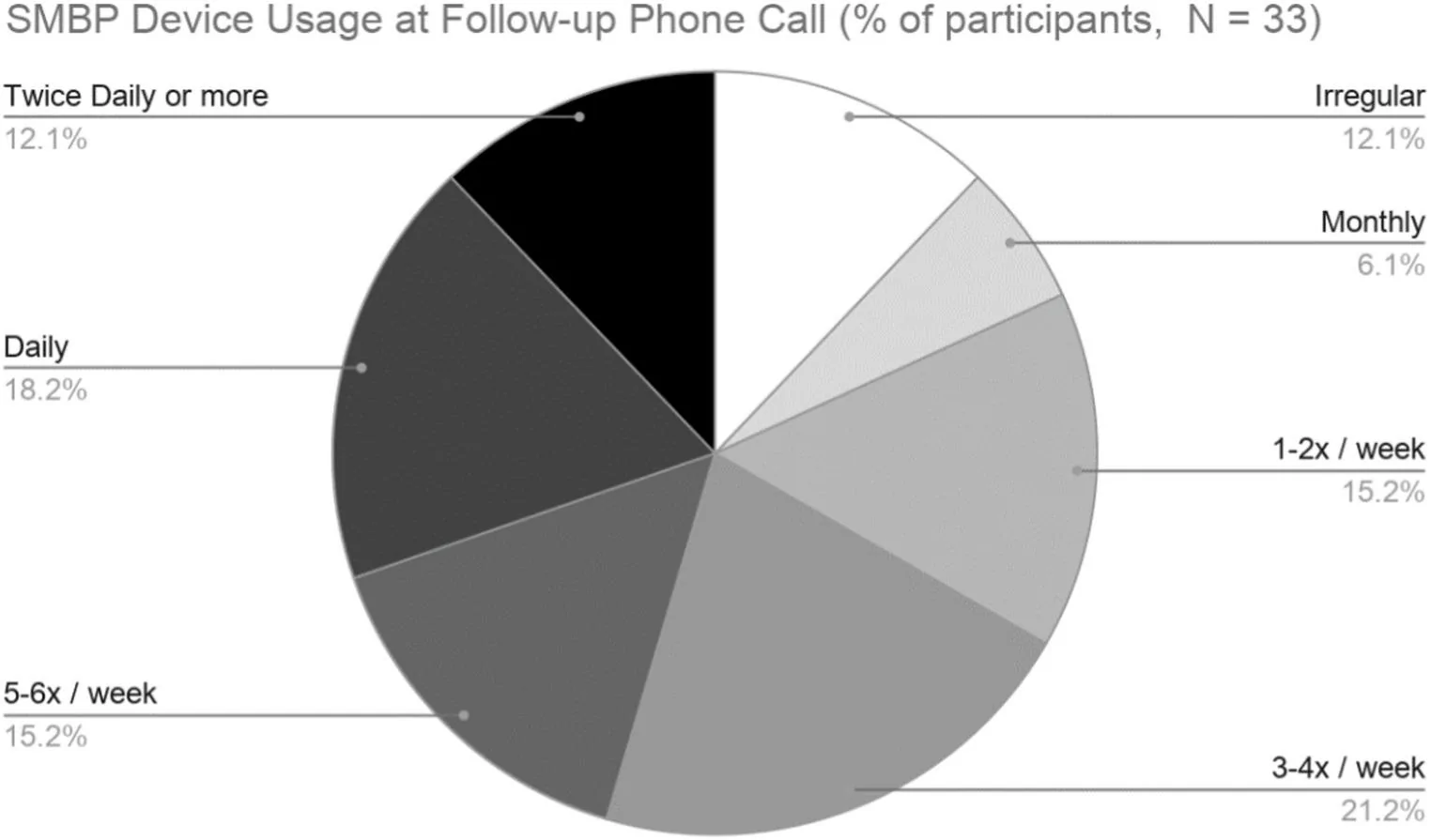
SMBP Device Usage at Follow-up Phone Call Among Reached Participants Using the Device (N = 33)

Regarding primary care follow-up, 21 (57%) of the 37 participants who answered a follow-up call reported contacting their PCP since the MHI event. Of those who specified further, 10 participants completed a recent PCP visit, 6 had an upcoming PCP visit, and 3 had contacted their PCP but had not scheduled an appointment. The remaining participants who had not contacted their PCP cited the following reasons: lack of time (*n* = 2), no insurance (*n* = 2), a strong relationship with and confidence in their PCP to attend to the matter in their subsequent appointments (*n* = 3), and it being too early for their yearly routine visit (*n* = 4). Three participants reported needing help from a community health worker or healthcare navigator to access primary care services.

Finally, during follow-up phone calls, participants were asked about their knowledge of hypertension and blood pressure management. When asked to define a *normal* SBP, 30 participant responses recorded had a median value of 120 mmHg. When asked to describe a *high* SBP, 31 participant responses recorded had a median of 145 mmHg. Participants were also asked to name reasons why it is important to treat hypertension. Of the 36 recorded responses, nine (25%) participants cited general good health, and 22 (61%) provided specific conditions, including stroke and heart attack.

## Discussion

HI HOPE is a response to a statewide need to improve hypertension control among those facing health disparities. Study participants reflected these disparities, as 80% of participants were Black or Hispanic, 43% did not have a prior hypertension diagnosis, 22% had known hypertension without being on antihypertensive medications, 21% were uninsured, and 34% did not have a PCP.

The high usage rate of SMBP (89%) among HI HOPE participants successfully reached by phone, with most participants reporting using it more than once, suggests the potential utility of such devices and reflects the participants’ curiosity about their BP and health in general. Our results are in agreement with prior studies. A survey by the University of Michigan found that 78% of the respondents whose clinicians instructed them to monitor their BP at home did so using SMBP [12]. Another prior study found that compliance, defined as using SMBP at least eight times in 12 months, was 74% [13]. Additional evidence shows that using an SMBP device alone reduces BP and improves medication adherence compared to standard care [8, 9]. Participants using the SMBP device in our study demonstrated engagement and blood pressure awareness.

The results of HI HOPE also demonstrate the challenges of attempting follow-up phone calls. While the study did not meet the ambitious prespecified feasibility threshold of successfully contacting at least 90% of participants with follow-up calls, it highlighted opportunities for improvement and provided instructive lessons integral to the design of the follow-up study called HI HOPE 2.

66% of participants were successfully contacted with a follow-up phone call after three attempts. Multiple factors are hypothesized to contribute to the difficulty in reaching one-third of patients by phone, including invalid phone numbers, calling when the participant is unavailable, or the time demands of a phone call on the participants. Ideas to mitigate these issues at the time of enrollment include ensuring a valid phone number is on file, asking participants for their preferred time of day for a follow-up call, and honoring that time to maximize the likelihood of a successful phone call. As was pointed out by one community partner, some in this community may have inconsistent cell phone plans due to socioeconomic factors; consideration could be given to connecting interested participants with Lifeline plans (“Obama phone”), which provides eligible Americans with a free cell phone [14].

The average time to a successful follow-up call was 36 days after the MHI visit, longer than the targeted 2 weeks. The delay in making phone calls was multifactorial. Because HI HOPE was unfunded, many team members were involved on a volunteer basis or had to prioritize other duties within MHI. The time needed to make follow-up calls was underestimated and proved to be a notable burden, as 15 (41%) participants required more than one phone call attempt. The coordination of this was further challenged by the need for phone interpreters for those participants who preferred a language other than English. MHI volunteers carried out such coordination without administrative support.

Two-thirds of participants had an established PCP. More than half of the participants who were successfully reached with a follow-up phone call had contacted their PCP between the MHI visit and the follow-up phone call. Among participants who did not contact their PCP, a variety of reasons were reported, including a recent PCP visit and confidence that the PCP would attend to the matter without needing to be contacted. Three participants expressed a need for assistance to connect with primary care services. This highlights an opportunity for follow-up calls in bridging the gap between MHI visits and primary care.

Follow-up calls also provided insight into the participants’ knowledge of hypertension. The majority of the participants were able to correctly articulate normal and high BP values and the potential consequences of uncontrolled hypertension.

HI HOPE was instructive in designing its follow-up study, HI HOPE 2, which has been funded by a UMN Program in Health Disparities Research grant and approved by the UMN IRB. Foundationally, the high rate of SMBP utilization in HI HOPE was reassuring and motivated the pursuit of this second study, which also includes distributing SMBP to individuals with elevated BP at screening. Given lessons learned about the need for dedicated administration and coordination to ensure timely follow-up with participants, we sought funding to provide salary support for this work. Furthermore, at enrollment, we are collecting preferred day and time for follow-up calls, as well as voluntary consent to be contacted by text message or email to coordinate the phone call, in an effort to increase the response rate. Additionally, HI HOPE 2 is a joint project with a nonprofit organization that serves the African American community and has collaborated with our MHI team on various health events, including health screening events. This organization, founded and operated by community leaders in North Minneapolis’ African American community, has deep ties throughout the community with nearly two decades of service. We believe that this collaboration will ensure the success of HI HOPE 2 and follow-up studies, from better-reaching participants for follow-up calls to ultimately addressing the hypertension disparity in this community.

As an unfunded feasibility study, HI HOPE was limited by its small sample size of 56 participants; therefore, findings must be interpreted cautiously with respect to generalizability. With supportive funding, HI HOPE 2 aims to enroll more participants (*n* = 100) and gather more specific information about barriers to primary care and other barriers to hypertension control facing the African American community. Moreover, we are formalizing the referral process to some local primary care clinics that can accept new patients and provide prompt, free, or low-cost care. We hope that this larger project will yield actionable strategies that organizations and healthcare systems can use to improve hypertension control in communities experiencing the highest disease burden.

## Conclusion

We found a high utilization rate of SMBP devices among individuals with elevated BP seen during mobile health visits. Contacting those individuals for follow-up by phone after the MHI visit is a complex and time-consuming process that warrants dedicated financial support.

## Data Availability

Raw data from this study is available from the corresponding author on reasonable request, following approval from the relevant data custodians.

## Abbreviations

NHANES: National Health and Nutrition Examination Survey
UMN: University of Minnesota
MHI: Mobile Health Initiative
BP: Blood Pressure
SBP: Systolic Blood Pressure
SMBP: Self-Monitoring Blood Pressure Device
PCP: Primary Care Provider
HTN: Hypertension
